# Neuropsychological deficits have only limited impact on psychological well-being in amyotrophic lateral sclerosis

**DOI:** 10.1007/s00415-021-10690-8

**Published:** 2021-07-02

**Authors:** Till Schrempf, Julia Finsel, Ingo Uttner, Albert C. Ludolph, Dorothée Lulé

**Affiliations:** Department of Neurology, University of Ulm, Oberer Eselsberg 45, 89081 Ulm, Germany

**Keywords:** ALS, Cognition, Well-being

## Abstract

**Objective:**

To investigate the association between neuropsychological deficits and psychological well-being in amyotrophic lateral sclerosis (ALS).

**Methods:**

Subjective (Schedule for the Evaluation of the Individual Quality of Life-Direct Weighting, SEIQoL-DW) and global quality of life (QoL; Anamnestic Comparative Self-Assessment, ACSA) as well as depression (ALS-Depression-Inventory, ADI-12) as indicators for psychological well-being were measured in 214 patients with ALS and correlated with neurocognitive performance assessed by the Edinburgh Cognitive and Behavioural ALS Screen (ECAS). Primary caregivers evaluated behaviour. Patients were classified to be cognitively (ALSci) or behaviourally impaired (ALSbi) according to Strong criteria.

**Results:**

ALSbi patients had poorer psychological well-being than patients without behavioural alterations, while the psychological well-being of patients with and without neurocognitive deficits was comparable.

**Conclusion:**

The study provides evidence that minor neuropsychological deficits do not interfere with psychological well-being of ALS in contrast to alterations on behavioural level. Thus, abnormalities in individual cognitive domains have limited relevance for the patients’ everyday life in comparison to the impact of behavioural alterations.

## Introduction

Amyotrophic lateral sclerosis (ALS) is a fatal neurodegenerative disease leading to death because of respiratory insufficiency within 3–5 years. The psychosocial adaptation model provides an explanatory approach for the fact that many ALS patients succeed in adapting to their new circumstances despite a fatal diagnosis [1]. The quality of life of ALS patients can be unexpectedly high; over time, it may be within the range of chronic ill patients without severe neurological impairment [2]. The quality of life is positively correlated to social support and negatively correlated to hopelessness, a sense of burden and suffering [3].

Neuropsychological deficits in ALS patients have been reported in up to 50% [4]. They affect either cognitive domains such as executive function or behavioural domains such as apathy and disinhibition. These deficits do not interfere with medical decision-making in ALS patients [5].

The psychological burden is not only on the patient but also on his or her caregivers [6]. The higher the physical limitation, the greater the burden for the relatives [7]. Improving the mental health of the caregiver seems to alleviate the patient’s distress [8]. Whereas behavioural alterations have been reported to negatively impact caregivers’ well-being [9], our understanding of whether neuropsychological deficits affect well-being is limited.

## Materials and methods

214 ALS patients visiting the in- and outpatient clinic in Ulm were included. They were diagnosed according to the revised El-Escorial-criteria (Table 1). Progressive bulbar palsy, primary lateral sclerosis, primary muscle atrophy, and flail arm syndrome belonging to the ALS spectrum have been included. Diagnosis of full-blown frontotemporal dementia (FTD) or lack of German language knowledge was excluded. Physical functioning was determined with the revised ALS Functional Rating Scale (ALS-FRS-R). Pathological laughter and crying was clinically defined by both, trained neurologists and psychologists. Further, patients were asked about pathological laughing or crying.

**Table 1:** Demographics and clinical data of participants

	Total records (*n* = 214)	ALSci (*n* = 88)	ALSbi (*n* = 26)	ALS (*n* = 70)	Statistics	
					*X*^2^	*p*
Age (years)	60.1 (12.5)	60.0 (16.0)	59.0 (21.0)	63.0 (19.0)	1.20	0.55
Education (years)	13.6 (3.3)	13.0 (2.0)	12.8 (5.8)	12.5 (5.0)	0.17	0.92
Gender (*n*)					5.07	0.08
Male	130	60	12	39		
Female	84	28	14	31		
ALS-FRS-R	38.71 (6.4)	41.0 (9.0)	37.0 (11.0)	40.0 (7.0)	3.51	0.17
PEG or NIV (%)	17.8	17.0	26.9	14.3	2.12	0.35
Duration since onset of symptoms (months)	28.13 (39.6)	15.0 (17.0)	18.5 (12.0)	14.0 (28.0)	0.67	0.72
Pathological laughter and crying (%)	14.0	13.6	11.5	21.4	0.19	0.91
Riluzole intake (%)	51.9	52.2	65.4	68.6	2.51	0.29
ECAS total score	107.2	98.9	112.1	113.3	44.47	< 0.01
ECAS-specific score	80.2	73.0	84.5	85.4	61.46	< 0.01
ECAS nonspecific score	27.0	25.9	26.5	28.1	5.72	0.06
ECAS behaviour score	0.39	0.2	1.5	0.1	81.10	< 0.01
	(*n* = 161)	(*n* = 64)				

Application of revised Strong criteria divided the study population into the groups of ALSci, ALSbi, and ALS

*ALSci* ALS with cognitive impairment, *ALSbi* ALS with behavioural impairment, *ALS-FRS-R* revised ALS Functional Rating Scale, *PEG* percutaneous endoscopic gastrostomy, *NIV* non-invasive ventilation

The German version of Edinburgh Cognitive and Behavioural ALS Screen (ECAS) [10] contains a cognitive screening and a behavioural assessment, filled in by the patients’ relatives. Patients were classified as either behaviourally (ALSbi) or cognitively (ALSci) impaired according to Strong criteria [11] or behaviourally and cognitively normal (ALS). Psychological well-being was assessed by means of the ADI-12, ALS-Depression-Inventory [12], the Schedule for the Evaluation of the Individual Quality of Life-Direct Weighting (SEIQoL-DW) [13], and the Anamnestic Comparative Self-Assessment (ACSA) [14], as measures of psychosocial adaptation.

ADI-12 is a short self-assessment screening questionnaire consisting of 12 items. ADI-12 has been validated for ALS patients [12]. Patients rate how much they agree with each statement on a 4-point Likert scale. ADI-12-Scores range from ‘0’ (best score) to ‘48’ (worst score). Clinically relevant depressive symptoms are indicated by scores above 28 [15].

The SEIQoL-DW is a self-assessment scale for subjective quality of life. Patients are asked to name five individual life areas which are relevant for their quality of life, and rate the importance of each area and their level of contentment with each area. SEIQoL-DW has a high internal validity in ALS patients [16].

The ACSA is a self-assessment scale to measure global quality of life. For ACSA, patients evaluate their current state with autobiographic episodes, which refer to the patient’s worst and best quality of life, respectively. These episodes are defined as endpoints ranging from − 5 to + 5 and the patient is asked to categorize the current QoL according to these endpoints.

The study was approved by the Ethics Committee of the University of Ulm (vote 19/12). All participants gave written informed consent to the study.

### Statistics

After calculating the Kolmogorov–Smirnov test to assess for normal distribution, Chi-square tests and Kruskal–Wallis tests were conducted to compare demographic and clinical characteristics. Mann–Whitney *U* tests were used to analyse cognitive performance and psychological well-being. Association between physical impairment and subjective quality of life (sQoL) was investigated by Spearman-Rho coefficient.

The significance level was adjusted at *p* = 0.05. Due to exploratory nature of the study, *p* values were not adjusted for the number of tests. Medians (Med) and interquartile ranges (IQR) are reported. The statistical analysis was performed with SPSS (Statistical Package for the Social Science) 24.

## Results

### Demographic data

Mean age of the study population was 60.1 years. Patients had 13.6 education years. Male-to-female ratio was 1.55:1. 28.1 months have passed since onset of symptoms and mean ALS-FRS-R was 38.7. 51.9% of the study population were medicated with riluzole. 14% showed pathological laugher and crying.

### Cognition

83.2% of the study population had an overall ECAS score above the cut-off [10]. The most prominent neurocognitive deficits were in the domains of language function (i.e., naming, comprehension, spelling; 25.2%) and verbal fluency (22.0%).

### Behaviour

Response rate of ECAS behavioural assessments was 75.2%. In 29.1% of the behavioural assessments, behavioural alterations of ALS patients were reported. Mostly (23.6%), a single domain was reported to be altered with apathy (15.2%) as the most common behavioural alteration. Patients who presented with impairments in both cognition and behaviour (ALScbi, *n* = 13) were excluded from further analyses for being a too small group.

### Psychological well-being

The median sQoL of SEIQoL-DW was 75.9%. ALS patients with neurocognitive deficits showed psychological well-being in the range of those without deficits. Physical restriction was not correlated with sQoL (Spearman-Roh: ALSci: *r* = 0.06; *p* = 0.59, ALSbi: *r *= 0.05; *p* = 0.83).

### Neurocognitive deficits and psychological well-being

**Fig. 1:**
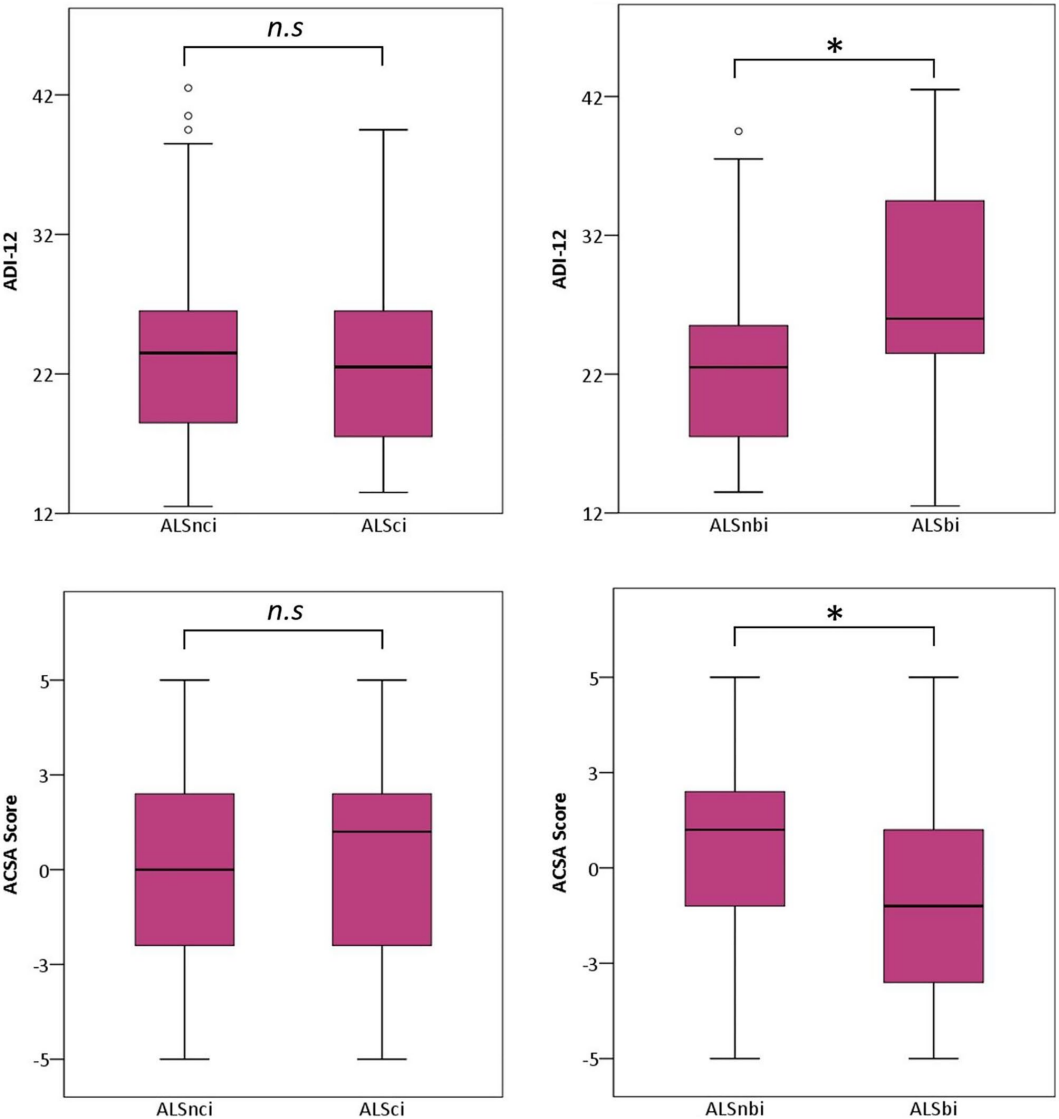
Comparison of psychological well-being between ALS patients with behavioural impairment (ALSbi) and without (ALSnbi) and between ALS patients with cognitive impairment (ALSci) and without (ALSnci). Behaviourally impaired patients showed poorer global quality of life (ACSA; *p *= 0.04) and higher depression (ADI-12; *p *= 0.04). *Indicated significant differences with *p *< 0.05. N.s. stands for not significantly

QoL and depression of ALSci patients did not differ from ALS patients without cognitive impairment (ADI-12: *U *=  − 0.84; *p *= 0.40; *r *= 0.09, SEIQoL-DW: *U *=  − 0.79; *p *= 0.43; *r *= 0.08, ACSA: *U *=  − 1.59; *p *= 0.11; *r *= 0.17) (Fig. 1).

Patients with depression (ADI-12 > 27) did not show neurocognitive deficits more frequently [*X*^2^(1) = 1.74; *p *= 0.19; *r *= 0.14], but had more often behavioural alterations [*X*^2^(1) = 9.24; *p* ≤ 0.01; *r *= 0.32].

### Behavioural alterations and psychological well-being

ALSbi patients scored lower in quality of life and higher in depression than ALS patients without behavioural alterations (ADI-12: *U *=  − 2.92; *p *= 0.04; *r *= 0.24, SEIQoL-DW: *U *=  − 1.30; *p *= 0.20; *r *= 0.25, ACSA: *U *=  − 2.04; *p *= 0.04; *r *= 0.16) (Fig. 1).

ALSbi patients did not perform worse in cognitive screening than patients without behavioural alterations (ECAS total score: *U *=  − 1.63; *p *= 0.10; *r *= 0.32, executive function: *U *=  − 1.56; *p *= 0.12; *r *= 0.31, memory: *U *=  − 0.67; *p *= 0.50; *r *= 0.13, visuospatial *U *= 0.78; *p *= 0.43; *r *= 0.15). In two domains, they performed significantly better (verbal fluency: *U *=  − 2.65; *p *= 0.01; *r *= 0.52, language: *U *=  − 3.09; *p *≤ 0.01; *r *= 0.61).

## Discussion

Patients with behavioural impairment showed poorer psychological well-being in comparison to patients without. Patients with cognitive impairment did not differ from patients without. A reason for the poorer quality of life of the ALSbi patients could be that the most common behavioural alteration was apathy as a negative symptom, known for its negative impact on quality of life [17].

This study shows that neurocognitive deficits have only little influence on subjective well-being, and therefore, expands previous findings of the impact of cognitive impairment on physical quality of life [18]. We hereby provide evidence that neurocognitive deficits and behavioural alterations show different relevance to everyday life and differently impact the psychological well-being of ALS patients. To subsume mild neurocognitive deficits under the headline of FTD might be misleading.

Psychological intervention has recently become an increasing focus in ALS. Our study shows that call for assessment and clinical management should not be restricted to neurocognitive deficits but also include aspects of managing behavioural impairment. Identifying premorbid personality structures that will particularly benefit from psychological intervention is a clinical challenge.

This study provides evidence that neuropsychological deficits in the sense of ALSbi or ALSci is not necessarily associated with better quality of life, interpreted as a loss of insight in the disease process. ALS/FTD patients show a lack of insight in the disease being a diagnostic criterion. However, it could be misleading not to differentiate between ALS/FTD patients and those without dementia [19]. As medical decision-making in ALS patients is independent from mild cognitive deficits, demands for very early preparation of patient’s advance directive to address the fact of high prevalence of neurocognitive deficits may be unjustified and may instead represent a restriction of patient autonomy [5]. Perception of ALS patients being in a quite good mood due to disinhibited behaviour contradicts the evidence shown. We therefore propose a more differentiated perception of neurocognitive deficits in ALS to improve patient clinical management.

### Limitations

Regarding physical limitation, the patient group examined was mildly restricted and examined at an early stage of the disease. Thus, no extrapolation for more advanced stage can be provided.

It is a limitation that screening instruments were used to assess cognition, but these provide similar results as extensive neuropsychological tests [20].

A shortcoming of the study is the cross-sectional investigation in the context of a clinic visit. Studies in which patients are longitudinally visited at home would overcome this bias. Also, causal relationships between neurocognitive deficits and psychological well-being cannot be explored by this cross-sectional approach. Furthermore, psychological well-being is a multidomain construct with depression and quality of life probably not covering the full range. Finally, due to exploratory nature of the study, we did not correct for multiple comparison (increasing the chance of false-positive results) which may reduce relevance of the work, so future research is needed to further explore relationships between neurocognitive deficits and psychological well-being.

### Conclusion

Based on the effect of neurocognitive deficits on the psychological well-being of ALS patients, the relevance of abnormalities in individual cognitive domains in everyday life should be critically considered.
